# Silicon photonics waveguide array sensor for selective detection of VOCs at room temperature

**DOI:** 10.1038/s41598-019-52264-9

**Published:** 2019-11-19

**Authors:** Ricardo Janeiro, Raquel Flores, Jaime Viegas

**Affiliations:** Department of Electrical Engineering and Computer Science, Khalifa University of Science and Technology, Masdar City Campus, Abu Dhabi United Arab Emirates

**Keywords:** Integrated optics, Optical sensors

## Abstract

We report on the fabrication and characterization of a volatile organic compound sensor architecture addressing common drawbacks of photonic integrated sensors such as reusability and specificity. The proposed sensor, built on a silicon-on-insulator platform and based on arrayed waveguide interference, has a chemically selective polydimethylsiloxane polymer cladding, which encapsulates the waveguides and provides an expandable and permeable low refractive index material. This cladding material acts as the chemical transducer element, changing its optical properties when in contact with specific volatile organic compounds, whose presence in the context of environmental and public health protection is important to monitor. The sensor operates at room temperature and its selectivity was confirmed by multiple tests with water, toluene, chlorobenzene, and hexane, through which the sturdiness of the sensor was verified. A maximum spectral shift of about 22.8 nm was measured under testing with chlorobenzene, at a central wavelength of 1566.7 nm. In addition, a sensitivity of 234.8 pm/% was obtained for chlorobenzene mass percent concentrations, with a limit of detection of 0.24%_m/m_. The thermal sensitivity of the sensor has been found to be 0.9 nm/°C.

## Introduction

Volatile organic compounds (VOCs) are ever-present in the natural world, playing an important role in plant-to-plant communication and plant-to-animals messaging. While most VOCs from natural and biological sources do not pose health or environmental concerns, the same does not hold true regarding anthropogenic sourced VOCs. Man-made volatile organic compounds are generally associated with smog, respiratory tract diseases, and damage to key organs and systems, such as liver, kidneys, and central nervous system. The health threat posed by VOCs is real, some being extremely toxic or leading to long term injuries^1^. In the current scenario of growing environmental and health concerns, the monitoring of VOCs' levels in indoor and outdoor air and water systems is increasingly relevant. Traditional methods for detection of VOCs are based on high-performance liquid chromatography^2^, gas-chromatography^3,4^, and mass spectrometry techniques ^5^. Despite being accurate and selective, these techniques are also expensive, time consuming and bulky, hampering their practical distribution and application for real-time air quality or water monitoring. Other fairly disseminated VOC-detection techniques include the use of metal-oxide-semiconductor (MOS) transistor-based sensors^6^ and micro-electrochemical systems (MEMS) sensors^7^, based on the electrical resistance or resonant frequency change when VOCs adsorb on the surface of a metal oxide or piezoelectric film, respectively. Those sensors address problems as test cost, equipment bulkiness, and the long laboratory processing times of conventional techniques. However, their operation requires high temperatures, around 200–400 °C, implying the usage of a heater for on-chip temperature control^8–10^, thus defeating the low power operation advantages of both MOS and MEMS technologies. Furthermore, selectivity to specific chemicals is limited.

Tackling the drawbacks affecting conventional VOC detection systems and techniques, we propose a silicon photonics integrated optical chemical sensor, which enjoys all the traditional advantages of optics, such as low cost, low power consumption, compactness and electromagnetic interference immunity, while combining the benefits provided by the micro and nanofabrication regarding size, scalability (high density chip integration) and potential for integration with the microelectronic industry under the CMOS fabrication technology^11–13^. Moreover, the proposed device effectively addresses two traditional issues affecting optical chemical sensors: limited chemical selectivity and sensor reusability. In label-free detection concepts the selectivity issue is commonly addressed through surface functionalization of the sensor. Although efficient when applicable, the functionalization adds an extra level of system complexity to both the modelling and the fabrication process. Reusability is also a strong impairment to practical and commercial deployment of integrated optical sensors in field applications, since conventional devices suffer from accumulation of particles in the sensing window used to expose the optical field to the analyte, with the resulting contamination affecting the device accuracy and reliability over time.

The proposed integrated Si photonics VOC sensor is based on parallel waveguide coupling and interference, and capably addresses the mentioned selectivity and reusability problems using Polydimethylsiloxane (PDMS) as the top cladding material. PDMS can be used as the cladding material of a silicon photonic sensor, acting as a selective sensing layer due to its selective swelling and chemical absorbing characteristics^14^. When in contact with some organic solvents, the PDMS swells and absorbs them, inducing a detectable change on the refractive index of the device’s top cladding. Effectively shielding the sensor from particle contamination and all other chemical components that do not react with it (e.g. water and methanol), the PDMS poses itself as a quite suitable transducing material for selective chemical detection of some VOCs of interest, like hexane and toluene. The use of PDMS as a selective sensing material has been demonstrated in both optical fibre^15,16^ and integrated optical platforms^17,18^.

Unlike prior work based on SiON-SiO_2_ substrates^19^, the selected silicon-on-insulator (SOI) platform, through its high refractive index (RI) contrast (n_Si_ = 3.47, ${{\rm{n}}}_{{\rm{S}}{\rm{i}}{{\rm{O}}}_{{\rm{2}}}}$ = 1.44 and n_PDMS_ = 1.40 at 1550 nm) allows for an extended range of measurable refractive indices, with no loss in sensitivity. Furthermore, due to the relatively large spectral distance between dips of the output spectrum, the proposed sensor allows for an extended range of unequivocally measurable refractive indices when compared with the majority of the conventional two-wave interferometers and resonators sensors, whose reduced free spectral range limits the maximum unambiguously detectable spectral shift^20^.

## Working Principle

**Figure 1:**
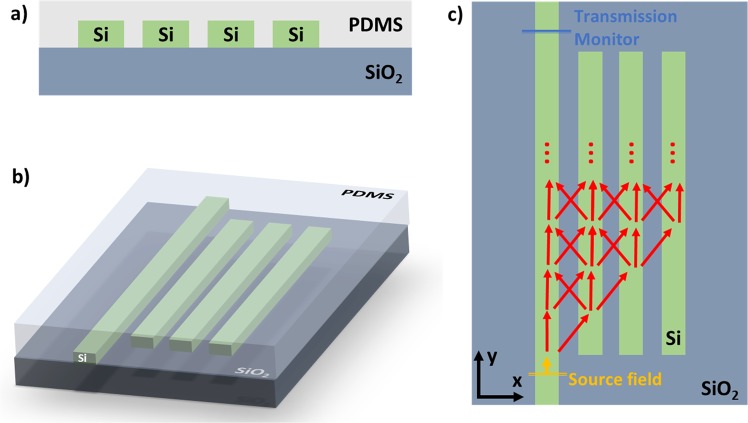
Diagram of the proposed concept: a sensor composed by a set of parallel Si waveguides on a SiO2 layer and covered by a PDMS cladding. Three views of the sensor are presented: (**a**) cross-section, (**b**) perspective view, and (**c**) top view, with the red arrows representing the light travelling through the device with multiple lateral coupling events.

A schematic of the device is presented in Fig. 1. The device is constituted by a set of evanescently coupled parallel waveguides. In the weak coupling regime, the modes of the individual waveguides are assumed to remain approximately unchanged, with the coupling solely affecting their amplitudes, leaving the field distribution and propagation constants unperturbed. Assuming the propagation along the *y* direction, the total electric field distribution of a structure constituted by an arbitrary number, *N*, of waveguides is given by coupled-mode theory^21^:

1 $$E(x,y,z)={a}_{1}(y){E}_{{a}_{1}}(x,z)+{a}_{2}(y){E}_{{a}_{2}}(x,z)+\cdots +\,{a}_{N}(y){E}_{{a}_{N}}(x,z)$$

where a_i_(*y*) are the propagation dependent amplitudes, and E_ai_(*x, z*) are the unperturbed modal field distributions of the *i-th* waveguide in the absence of neighbouring waveguides.

Considering coupling between first-neighbors, the amplitudes satisfy the coupled-mode equations:

2 $$\frac{{\rm{d}}}{{\rm{d}}y}{a}_{i}(y)=j{K}_{i-1,i}\,{a}_{i-1}+j{\beta }_{i}\,{a}_{i}+j{K}_{i,i+1}\,{a}_{i+1}$$

where ${\beta }_{i}$ is the propagation constant of the *i-th* waveguide and ${K}_{i,i+1}$ is the coupling coefficient between the *i-th* and the *(i+1)-th* waveguides. In matrix form, the coupled-mode equations take the form of

3 $$\frac{{\rm{d}}}{{\rm{d}}y}[\begin{array}{c}{a}_{1,v}\\ \vdots \\ {a}_{N,v}\end{array}]=j{\bf{M}}{\boldsymbol{[}}\begin{array}{c}{a}_{1,v}\\ \vdots \\ {a}_{N,v}\end{array}{\boldsymbol{]}}$$

where **M** is a tridiagonal matrix whose main diagonal is filled by the individual propagation constants of each waveguide, and the diagonals above and below the main diagonal are filled with the coupling coefficients between first neighbors.

The longitudinal amplitude modulation parameters of the waveguide propagating mode fields are given by a linear combination of orthogonal solutions of the form^21^:

4 $$[\begin{array}{c}{a}_{1,v}\\ \vdots \\ {a}_{N,v}\end{array}]=[\begin{array}{c}{A}_{1,v}\\ \vdots \\ {A}_{N,v}\end{array}]{e}^{j{\beta }_{v}y}$$

where *β*_*v*_ and vector **A**_**v**_ are, respectively, eigenvalues and eigenvectors of the matrix used to express the coupled mode equations of an *N* waveguide coupler in matrix form **M**. The general solution of the homogeneous linear system of the coupled mode differential equations is the linear combination of the above eigensolutions, with constants of proportionality defined by the initial value of vector **a**, [**a**(0)].

When the PDMS swells and absorbs certain chemicals, it changes its refractive index. As the top cladding index changes, so do the propagation constants and the coupling coefficients. This way the eigenvalues and eigenvectors of the system change. As such, the system general solution changes, resulting in an alteration of both the characteristic electric field pattern distribution over the entire structure and its spectral output.

### Device design and simulation

The studied device is based on 450 nm-wide and 220 nm-thick Si strip waveguides on a buried silicon oxide layer with a PDMS upper cladding. The waveguides are single mode in the spectral window of the selected source (1500–1600 nm).

A directional coupler composed by *N* waveguides has *N* supermodes for both the characteristic quasi-TE and quasi-TM polarizations, each with a corresponding field distribution. The total field propagation, for each polarization, is governed by the interference originated by the superposition of those *N* supermodes, each having different propagation constants and effective refractive indices.

**Figure 2:**
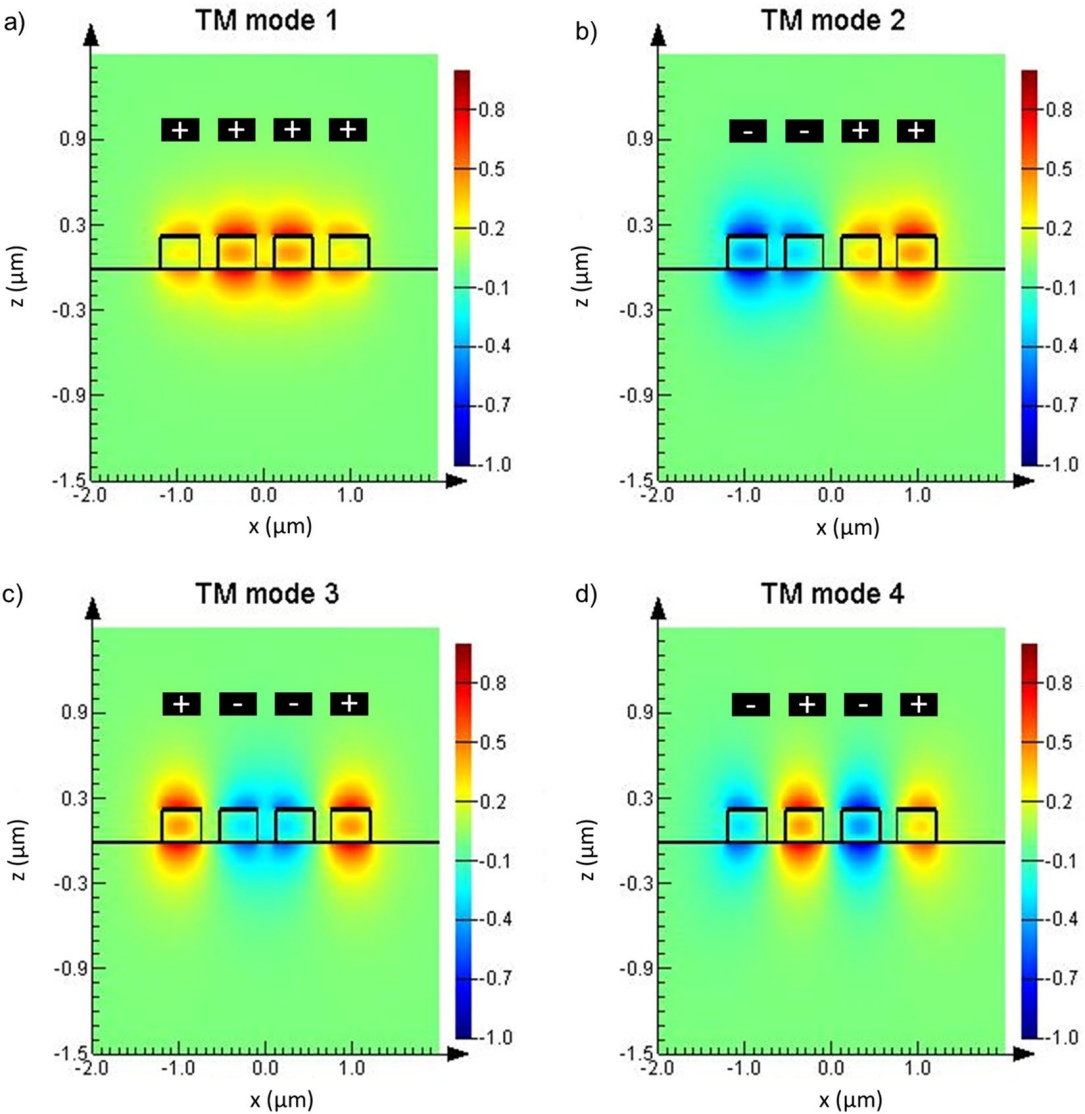
Electric field supermodal distributions for TM (*E*_*z*_) polarization for the first 4 supermodes.

The quasi-TM electric field modal distribution corresponding to a 4-parallel waveguide coupler is shown in Fig. 2. Modes 1 and 3 are symmetric modes, shown in Fig. 2(a,c), respectively, while modes 2 and 4 are anti-symmetric modes, Fig. 2(b,d), respectively. The TM mode plots show the dominant field component *E*_*z*_.

Compared with TE, the lower confinement of the TM polarization and its increased mode overlap with the top cladding gives it a higher dimensionless device sensitivity *S*_*h*_:

5 $${S}_{h}={\rm{d}}{n}_{eff}/{\rm{d}}{n}_{c}$$

where *n*_*eff*_ is the effective index of each supermode and *n*_*c*_ is the cladding index. Indeed, considering supermode 1 for illustrative purposes, the mode’s power percentage overlapping with the cladding region is 13.5% for TE and 30.5% for TM. For that reason, all simulations and results presented and discussed hereafter are based on TM polarized light.

The multiparametric study of the characteristic transmission and typical field propagation of the proposed structure was simulated by Finite-Difference Time-Domain method using Lumerical 3D FDTD software. The light was injected at the leftmost waveguide, with propagation along the *y* direction. The positions of the source and the transmission monitor are depicted in Fig. 1(c). The transmission plots were obtained from simulations in the wavelength range from 1500 nm to 1600 nm, whereas the intensity propagation plots correspond to a source wavelength of 1550 nm.

**Figure 3:**
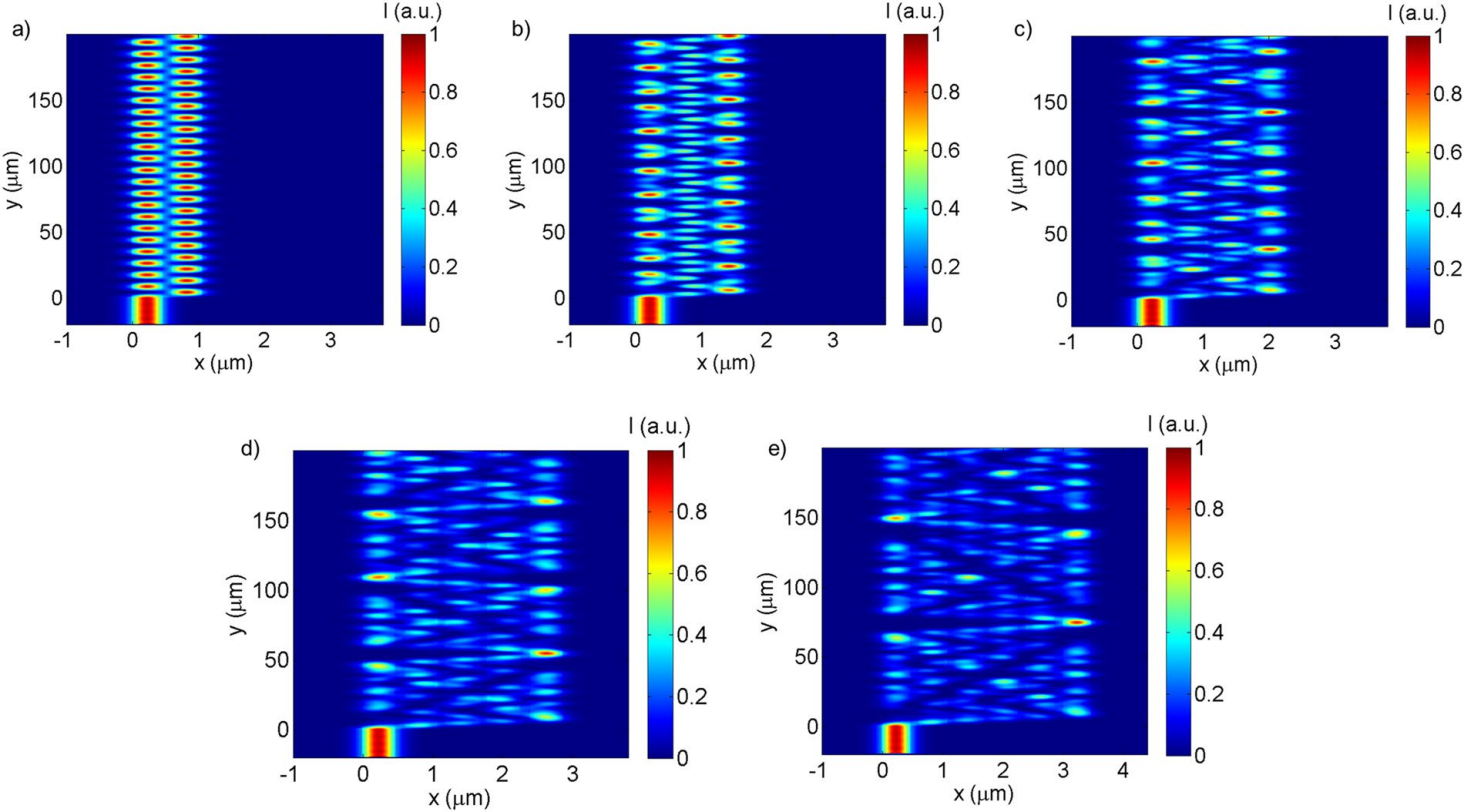
TM interference patterns of devices with the number of parallel waveguides ranging from 2 to 6 (constant gap between waveguides of 150 nm).

For a 200 µm long device, the field propagation pattern dependence on the number of waveguides is presented in Fig. 3, where the gap between waveguides was kept constant at 150 nm. The 2-waveguide device is a basic directional coupler, shown in Fig. 3(a), exhibiting the expected total output transfer between two similar waveguides and the corresponding sinusoidal evolution of the light intensity in each waveguide along the direction of propagation. With the increase of the number of waveguides, Fig. 3(b–e), the number of events of power transfer between waveguides increases, effectively increasing the light travel distance and consequently increasing the interaction between the propagating electromagnetic field and the chemically sensitive cladding. Likewise, increasing the number of waveguides also increases the period of the interference pattern along the direction of propagation, with the advantage of leading to a broader range of possible characteristic output patterns due to the increased complexity of the propagating field pattern.

**Figure 4:**
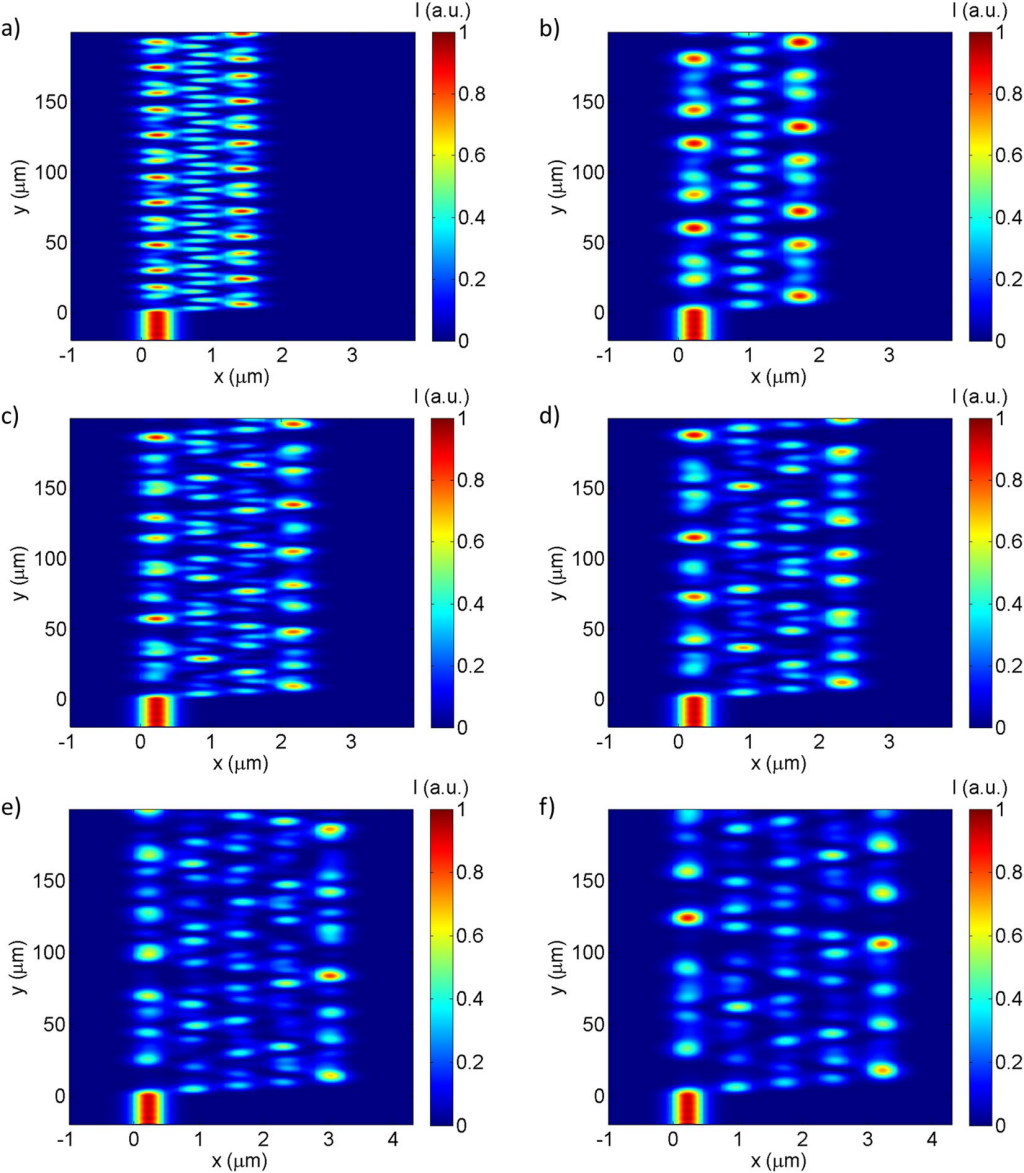
TM interference patterns of devices with 3 parallel waveguides, (**a**) 150 nm and (**b**) 300 nm apart each other; with 4 parallel waveguides, (**c**) 200 nm and (**d**) 250 nm apart each other; 5 parallel waveguides, (**e**) 250 nm and (**f**) 300 nm apart each other.

**Figure 5:**
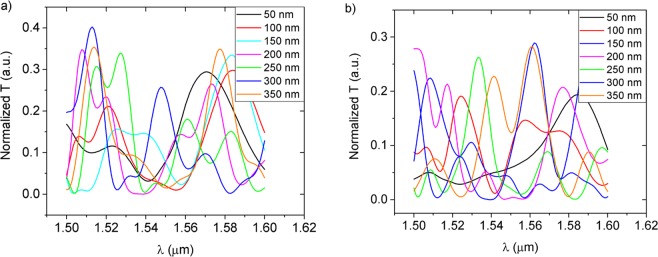
Simulated TM transmission spectra at the output port: (**a**) device with 4 parallel waveguides; (**b**) structure with 6 parallel waveguides.

The influence of the gap dimension, between consecutive waveguides, on the behaviour of the device was also studied. For a fixed number of waveguides, the distance between them was varied from 50 to 350 nm with steps of 50 nm. As the distance between waveguides increases, the coupling coefficient between waveguides decreases, the coupling length increases, and the interference pattern is stretched along the propagation direction, as can be seen in Fig. 4(a–f). The spectral responses, presented in Fig. 5, and their multiparametric evolution evidence a strong non-linearity and high sensitivity to design parameters, highlighting the flexibility and tailorable character of the device´s output spectrum. Such adjustability broadens the range of possible applications and device specifications, ranging from high precision measurements at a certain wavelength, to broader bandwidth applications for a wider range of detectable refractive index, or even hybrid devices combining high-RI-sensitivity/narrow-range regions with lower-RI-sensitivity/wider-range sections in the same sensor for multiplexed applications.

The choice of the fabricated device parameters obeyed some considerations: a) the challenges of fabricating nominal gaps inferior to 150 nm due to electron-beam lithography proximity effects in the long coupling regions and the difficulty of reproducibly filling small gaps with PDMS; b) devices with a higher number of waveguides have a longer self-imaging period, increasing their refractive index dynamic range and exhibiting a higher number of characteristic waveguide power distributions, useful in case of interrogation through pattern projection; c) devices with less than four waveguides have a range of patterns fairly limited, thus have been excluded; d) devices with more than four waveguides entail a comparatively more challenging electron beam lithography process due to the necessity of further exposure dose control and proximity-effect correction.

## Device Fabrication

**Figure 6:**
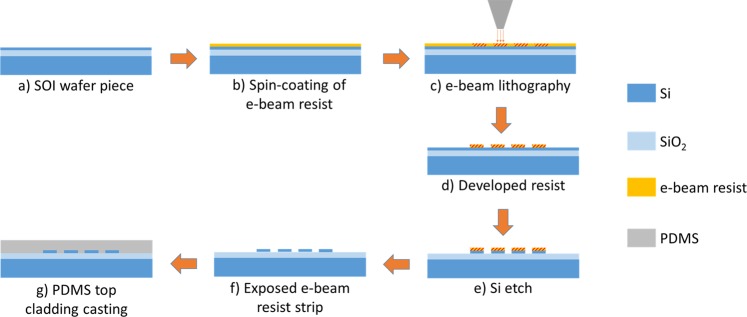
Device fabrication sequence.

The chip fabrication process flow is summarized in Fig. 6. An 8 × 8 mm^2^ SOI wafer piece was used, Fig. 6(a), and the wafer stack was composed by a 220 nm-thick Si device layer and a 3 μm-thick SiO_2_ buried layer on a 675 µm Si handle layer.

The parallel waveguide structure was patterned through electron-beam lithography, depicted in Fig. 6(b–d). Prior to the e-beam lithography, the sample was coated with ma-N 2403 for 45 s at 3000 rpm, followed by a bake at 90 °C for 60 s.

The design width of the waveguides was set at 490 nm in order to achieve a 450 nm resist width after development and the nominal dose was set at 130 μC /cm^2^. However, in order to tackle the proximity effect in the region with high density of features, a non-uniform dose distribution was applied to the design: 100% of the dose was applied to the outer waveguides, but only 80% to the inner ones.

Following the lithography and development of the resist (50 s in ma-D 525 developer), a fluorine-based reactive ion etch (RIE) of the Si layer transfer red the pattern to the wafer stack, Fig. 6(e). The etching parameters were as follows: 50 W of RF power, 100 W of ICP power, base pressure of 1 Pa, 10 sccm of SF_6_, 35 sccm of CHF_3_, He back pressure of 2 kPa, for a duration of 90 s. The etching was followed by acetone stripping of the exposed e-beam resist, Fig. 6(f).

**Figure 7:**
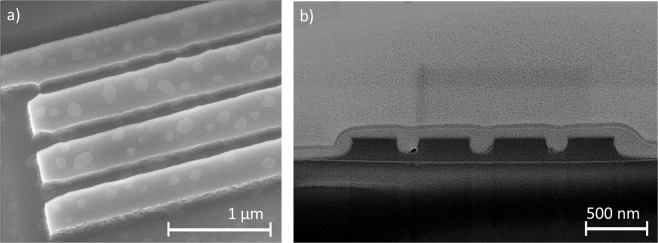
Scanning electron microscope images of the etched waveguides prior the PDMS coating: (**a**) isometric view; (**b**) cross section of the device.

Scanning electron microscope images of the etched waveguides are shown in Fig. 7. The fabricated waveguides are narrower than designed, despite the correct dimensions of the exposed resist. The exposed pattern constituted of designed 450 nm-wide waveguides and 150 nm gaps, once etched, resulted in a device with approximately 370 nm-wide outer waveguides, 410 nm-wide inner waveguides, and gaps of approximately 190 nm. This is due to etch loading effects. Higher pattern transfer fidelity can be obtained by keeping the etch density constant using fill structures but at the expense of a longer electron-beam lithography writing time.

In the final fabrication step, Fig. 6(g), the device was coated with PDMS (Sylgard® 184 Silicone Elastomer in a 10:1 weight ratio of base polymer to curing agent), having been dropcasted over the chip and submitted to a degas cycle to ensure a bubble-free device top cladding. The total effective area of the sensor was 426 μm^2^.

## Results and discussion

### Experimental setup for optical characterization

The characterization of the devices was carried out in a typical experimental setup for photonic chips testing. A C/L-band tuneable laser (Agilent 81600B Tuneable Laser Source) was used as light source. Both the light source and the optical power sensor (an InGaAs Agilent 81636B detector) were housed in an Agilent 8164B mainframe. The light was guided from the light source, passing through a manual fibre-polarization-controller (Thorlabs FiberControl FPC-2), coupled to the chip and collected by a pair of commercial lensed fibres (Nanonics SM 1300–1500 nm, 2 μm spot size, 4 μm working distance), and guided back to the photodetector.

The chip and the lensed fibres both sat on a set of *xyz* translation stages to allow proper chip-to-fibre alignment. All measurements were performed under TM illumination.

### Device characterization

To fully understand the influence of the PDMS on its performance, the sensor was characterized at room temperature (about 23 °C) with and without the polymeric top cladding.

**Figure 8:**
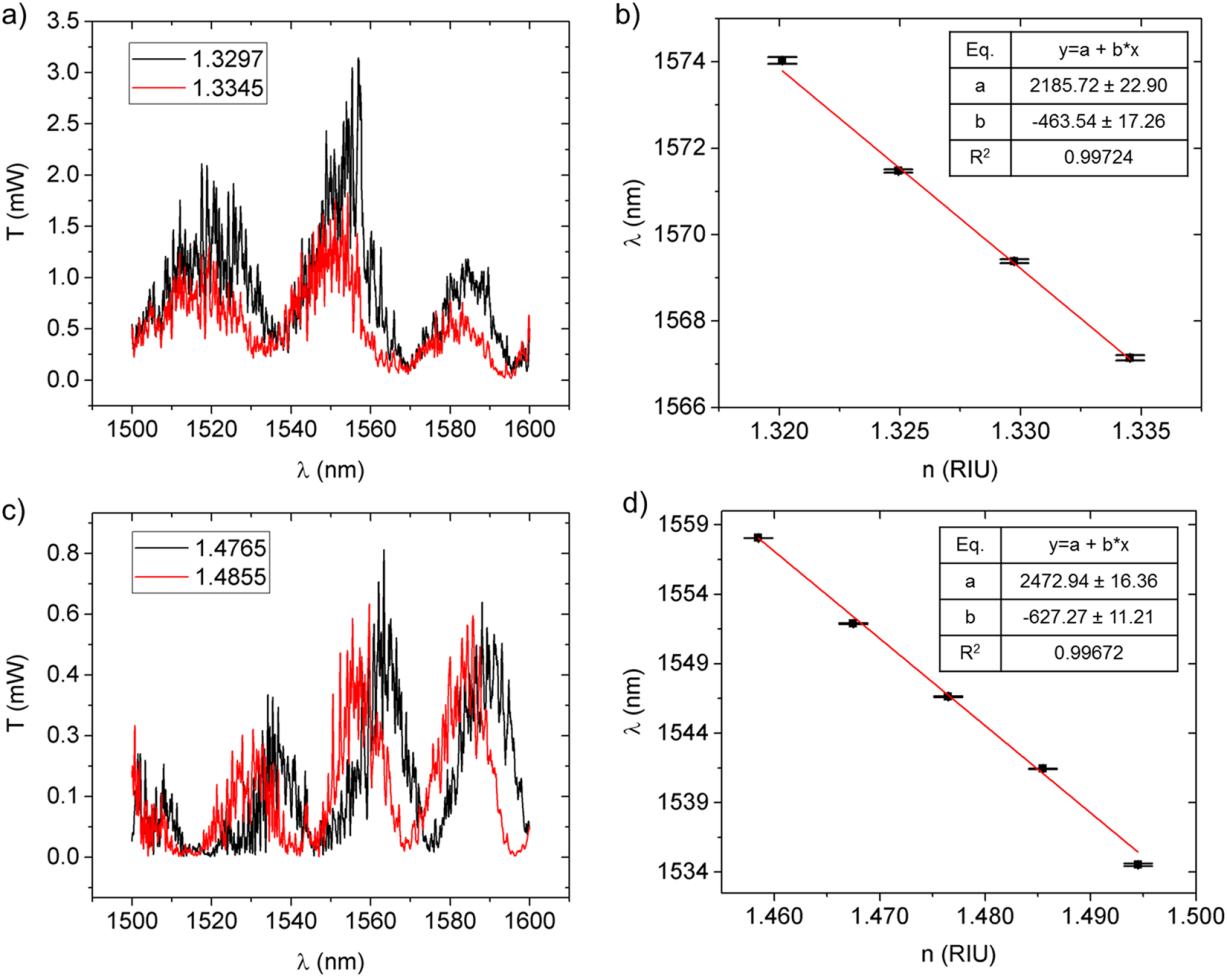
Spectral output of sensor and spectral shift during testing with environmental RI of: (**a**) 1.3297 and 1.3345; and (**c**) 1.4765 and 1.4855. Dependence of the spectral position of one of the dips in (**a**,**c**) on the environmental refractive index for (**b**) set A and (**d**) set B of Cargille liquids, respectively.

The characterization of the sensor fully exposed to the surrounding environment, i.e., without the PDMS top cladding, was performed with the use of two sets of refractive index calibration liquids (Cargille). The experimental results are shown in Fig. 8. This test yielded a sensitivity of −463 nm/RIU for an environmental refractive index ranging from 1.3201 to 1.3345 (Cargille liquids set A), and −627 nm/RIU for refractive indices ranging from 1.4585 to 1.4945 (Cargille liquids set B). All refractive index values mentioned in this manuscript correspond to a RI at a wavelength of 1550 nm.

The limit of detection (LOD), characterized as the minimum detectable variation of the environmental refractive index, depends on both the sensitivity of the device and the minimum spectral shift unequivocally attributed to index variations:

6 $$LOD=\frac{Minimum\,unequivocal\,shift}{Sensitivity}$$

Considering the minimum unequivocal detectable shift to be three times the standard deviation of a set of measurements obtained from 20 sequential wavelength scans, the LOD was determined to be 5.0 × 10^−4^ RIU for the lower range of refractive indices and 4.5 × 10^−4^ RIU for the upper range.

**Figure 9:**
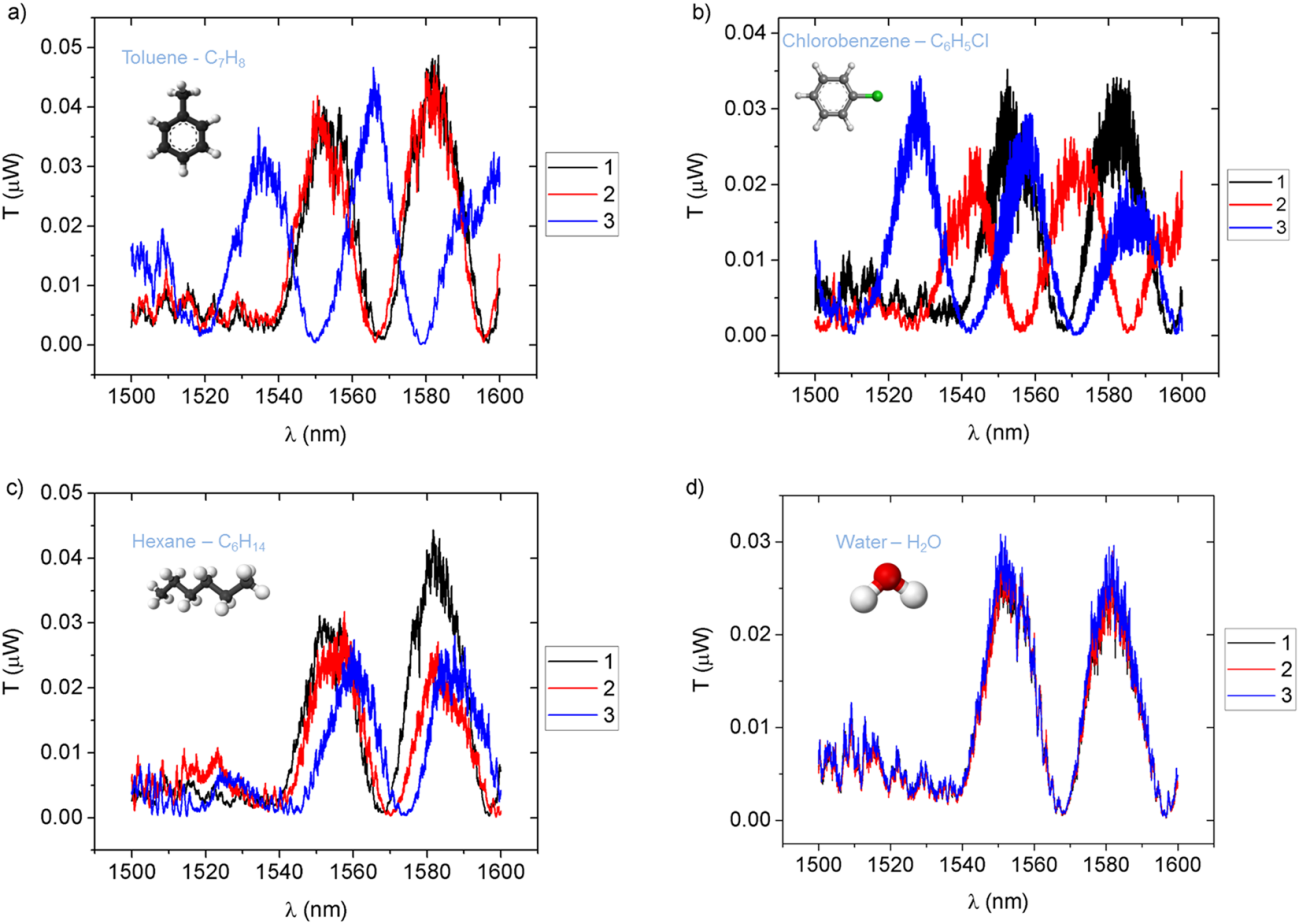
TM spectral response of the device upon contact with: (**a**) toluene; (**b**) chlorobenzene; (**c**) hexane and (**d)** water. Scans identified by numerals represent sequential measurements when the sensor is in contact with different substances. Scan 1 (black trace) corresponds to the dry state of the sensor, scan 2 (red trace) represents an intermediary state taken shortly after the device was subjected to the target chemical, and scan 3 (blue trace) shows the maximum wavelength shift of the spectral signature of the device for each VOC. In d), no shift was observed.

The performance, selectivity and reusability of the PDMS cladded device under chemical testing was characterized with deionized water (DI water) and several pure VOCs, namely toluene, hexane, and chlorobenzene in their liquid form. For each analyte, a 2.0 μL droplet of pure chemical was dispensed, which was large enough so that the chip surface was totally covered. The characterization of the device under VOC stimuli consists on sequentially performing wavelength scans, starting from the moment the liquid is brought in contact with the chip. The TM spectral responses are presented in Fig. 9 for (a) toluene, (b) chlorobenzene, and (c) hexane. Scan 1 (black trace) corresponds to the dry state of the sensor, scan 2 (red trace) represents an intermediary state taken shortly after the device was subjected to the target chemical, and scan 3 (blue trace) shows the maximum wavelength shift of the spectral signature of the device for each VOC.

Although both toluene^22^ (n_toluene_ = 1.4778) and chlorobenzene^23^ (n_chlorobenzene_ = 1.4928) have a refractive index within or in close proximity to one of the refractive index intervals tested with the Cargille liquids (1.46 to 1.49), the comparison between the spectral output of the sensor during the tests with the solvents (sensor with PDMS) and the Cargille liquids (sensor without PDMS) highlights the change in the sensing mechanism introduced by the PDMS cladding. Acting as a transducer element and reducing unwanted contamination of the sensor, the PDMS shields the waveguides from direct contact with the analyte. When the PDMS swells due to interaction with certain solvents, its refractive index decreases with the expansion. The combined effect of the PDMS swelling plus the solvent permeation leads to a cladding refractive index change: the cladding RI becomes a weighted average between the RI of the swollen, less dense PDMS and the refractive index of the permeating solvent. As such, the difference in RI between the claddings explains the different spectra of the sensor with and without PDMS for the same environmental RI. In both cases the spectral distance between any two consecutive output dips is always larger than 24 nm, allowing the unambiguous identification of relatively large spectral shifts.

When tested with toluene, Fig. 9(a), the spectral response blue shifts, i.e., it consists of a spectral shift towards shorter wavelengths. Accounting for the solvent permeation effect on the PDMS, the maximum shift is not expected to be achieved instantaneously and that assumption is confirmed by the intermediary scan 2 shown in Fig. 9(a). The maximum wavelength shift for toluene is approximately 18.0 nm for the dip around 1566.6 nm.

For chlorobenzene, as with the case of toluene, a blue shift is identifiable as can be seen in Fig. 9(b). The maximum amplitude of such shift is 22.8 nm, which is the largest for the VOCs tested.

Unlike the results described for toluene and chlorobenzene, when in contact with hexane a red shift (towards longer wavelengths) is identifiable from the successive spectral scans. This opposite behaviour was expected since it mimics the relative distribution of the refractive indices of the test chemicals: both toluene^22^ (n_toluene_ = 1.4778) and chlorobenzene^23^ (n_chlorobenzene_ = 1.4928) have a refractive index higher than the PDMS index, while hexane has a refractive index smaller than the top cladding^24^ (n_hexane_ = 1.3854). A maximum shift of about 6.3 nm was measured, the smallest for the set of reported VOCs.

In order to attest the selectivity of the PDMS-covered device, a test with deionized (DI) water was conducted. Since water induces no swelling in the PDMS, no spectral changes were expected from such test. The measurements are plotted in Fig. 9(d) and have confirmed the sensor’s insensitivity when in contact with water, as no spectral shift between dry (scan 1) and wet (scans 2 and 3) measurements was verified. This test confirmed the selectivity of the device as it only detects chemicals that cause PDMS swelling, which includes, but it is not limited to, non-polar and weakly polar solvents^15,25^. The insensitivity to water has the added advantage of making the sensor immune to relative humidy ambient values, thus increasing the robustness of the presented sensor. Furthermore, the role of PDMS in adding chemical selectivity to the sensor is highlighted when the DI water scans are compared with the measurements of the uncladded sensor (without PDMS). The comparison between the spectral shifts obtained during testing of the uncladded sensor with set A of the Cargille liquids, presented in Fig. 8(a,b) (RI values around the refractive index of water^22^ (n_water_ = 1.3154)) and the absence of any spectral change for the PDMS covered device during testing with DI water further underlines the chemical selectivity factor enabled by the PDMS.

A fixed wavelength characterization of the device was performed in order to analyse its dynamic behaviour as a function of time, during which the power output is monitored at while the VOC is brought into contact with the chip surface and allowed to dry afterwards. These measurements were performed at a fixed wavelength selected to correspond to a spectrum region of steep slope and low noise amplitude, assuring the operation of the sensor in its most sensitive and accurate region. For all tested VOCs a wavelength of 1563.7 nm was chosen. In addition, measurements at 1572.0 nm were also performed when testing the device with hexane.

**Figure 10:**
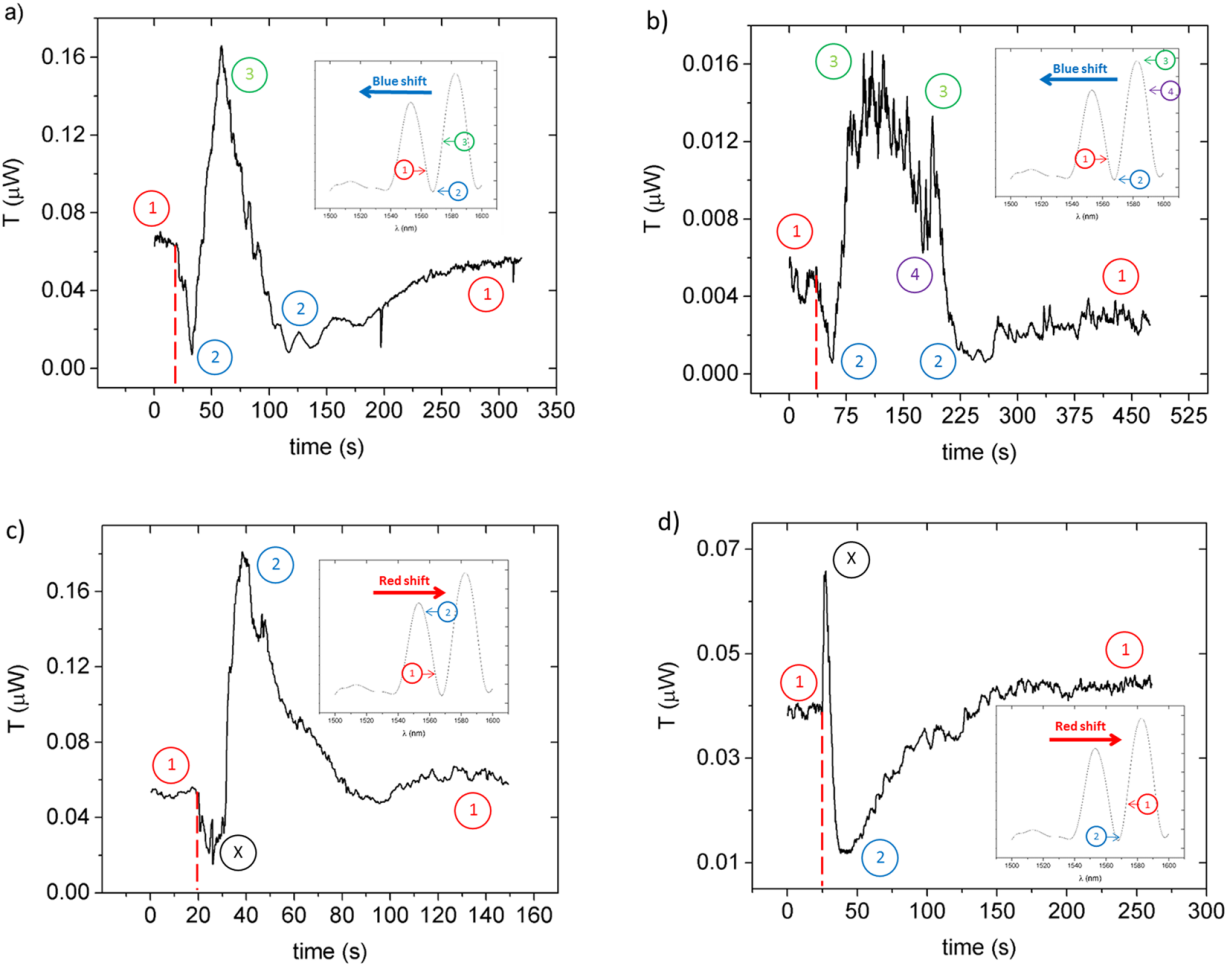
Fixed wavelength dynamic output evolution during the wetting and drying of the chip with: (**a**) toluene at 1563.7 nm; (**b**) chlorobenzene at 1563.7 nm; (**c,d**) hexane at 1563.7 nm and 1572.0 nm respectively. Insets illustrate the TM spectrum of the device as well as the correspondent shift direction that each chemical induces, represented by either a red or a blue arrow.

The experimental results are shown in Fig. 10. The output power is monitored for a period of time before placing the VOC in order to establish a baseline corresponding to the dry state. The dashed vertical red lines represent the time when the VOCs are dropped onto the chip surface. For all the tested VOCs, the sensor reacts within 200 ms from the moment it is subject to the VOC.

The insets in Fig. 10 show a representation of the output spectrum of the sensor, the direction of the shift induced by the solvent (indicated by a blue or red arrow), and the spectral positions of points ①, ②, ③, and ④, whose relevance is explained in detail hereafter.

The dynamic behaviour under toluene testing is shown in Fig. 10(a). The starting point is indicated by ① and the toluene is placed onto the chip at around 19 seconds into the experiment timeline, represented by the red vertical dashed line. A reduction in transmitted power occurs when toluene is placed onto the chip meaning that the spectrum moves towards smaller wavelengths, which agrees with the blue shift shown in Fig. 8(a). Due to the large spectral shift induced by toluene, the output dip moves past the reference wavelength (1563.7 nm) leading to the observed minimum output power, ②, followed by a rise in power to its maximum value at ③. The spectral positions of ①, ②, and ③ are depicted on the inset of Fig. 9(a). As the toluene evaporates and the PDMS contracts back to its original state, the spectrum starts to move backwards, until it restores to its initial position, ①. Analysis of the results shows that the time past from the initial stimulus up to the moment when the maximum spectral displacement is achieved, i.e. the sensor’s response time, is approximately 40 seconds. A naturally longer recover time is observed as it depends on the evaporation rate of the target VOC.

The dynamic response of the device under chlorobenzene stimulus, shown in Fig. 10(b), is also in agreement with the analysis of the consecutive wavelength scans. The initial decrease in transmitted power from ① when chlorobenzene is placed onto the chip, at around 35 seconds into the experiment timeline, corresponds to the aforementioned blue shift of the spectrum. The spectral shift induced by chlorobenzene is large enough to move both dip, ②, and the peak, ③, respectively seen at circa 1566.6 nm and 1582.0 nm, past the reference wavelength of 1563.7 nm. This corresponds to the output power sequentially passing through an absolute minimum, ②, followed by an absolute maximum, ③, and reaching a relative minimum, ④, after which the chlorobenzene starts evaporating and the sensor recovers to its dry state. Although in Fig. 9(b) the power output at the spectral points ① and ④ is distinct, the proximity in power output value between moments represented by ① and ④ in Fig. 10(b) is due to drift of the stages where the lensed fibres were mounted, that led to misalignment thus decreasing the power coupled into the chip. This statement is corroborated by the difference in output power of the initial and final dry states of the sensor, both labelled by ①. The sensor’s response time when submitted to chlorobenzene is about 150 seconds, which is significantly higher than for toluene but understandable given the larger spectral shift induced by the toluene.

The dynamic behaviour of the device when tested with hexane is consistent with the spectral shift towards longer wavelengths seen in Fig. 9(c) as the output power increases from ① to ②, which can be seen in Fig. 10(c). This phase is followed by a device recovery phase as the VOC dries and the PDMS contracts back to its original state, ①. The hexane experiment shows a response time of 19 seconds from the moment the liquid contacts the chip. However, there is a transient variation marked by Ⓧ in Fig. 10(c) corresponding to an initial power drop not in accordance with the red shift observable in Fig. 9(c). To confirm that such power variation was not a random measurement fluctuation, another dynamic measurement at a different reference wavelength was conducted. Indeed, further measurements at 1572.0 nm, shown in Fig. 10(d), display the same behaviour in which the sensor undergoes a fast blue shift, marked by Ⓧ, before resuming to its expected red shift towards the point ②. Since that measurement was realized at a reference wavelength higher than the dip, and considering the expected red shift, the output power should drop initially. However, just like in Fig. 10(c), right after the placement of hexane over the chip, the output power of the device evolves in the opposite direction for a brief period of time before proceding to the expected behaviour. This is believed to be a temperature effect in which the placement of the VOC cools down the chip, and such assumption is supported by the temperature study of the device.

The temperature characterization of the PDMS covered device was performed by monitoring the minimum of the dip centred at 1568.0 nm while the temperature of the chip was raised about 10 °C and subsequently lowered back to room temperature. To enable the measurement, the chip was placed on a Peltier element. A thermocouple was mounted in contact with the Peltier to properly monitor the temperature. Between temperature points, a stabilization waiting time was respected, larger than the stabilization time of the thermocouple to accommodate for the different thermal conductivities involved and ensure the thermal equilibrium of the entire system (thermocouple and chip with PDMS).

**Figure 11:**
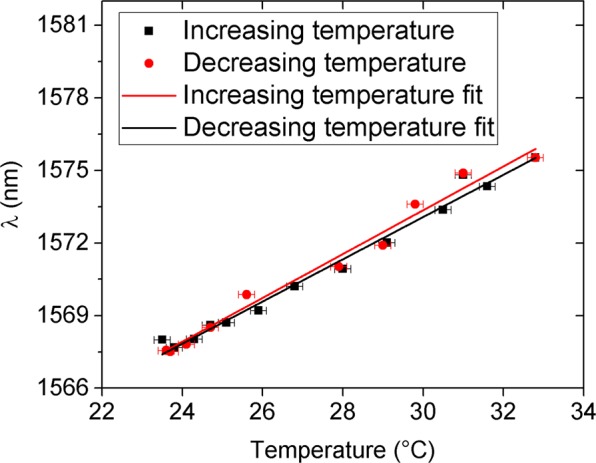
Temperature dependence of the monitored dip position (PDMS covered device).

The measurement results can be seen in Fig. 11. Upon heating up, a spectral shift towards the longer wavelengths was observed. The linear fits of both rise and fall in temperature have slopes of 0.89 nm/°C and 0.92 nm/°C respectively. The characteristic spectral shift under temperature variations explains the transient peak and dip marked by Ⓧ in Fig. 10(c,d). Indeed, in such plots, the presence of that sharp variation immediately after the liquid contacts the chip suggests a transient blue shift of the spectrum, which could be caused by a sudden negative temperature change since it translates into a shift towards shorter wavelengths. Such drop in temperature is caused by the liquid evaporation process. The effect of the temperature change, although present in all the VOCs experiments, only became evident in the hexane measurements due to the red shift this compound induces, while the characteristic blue shift of the other tested chemicals masks the shift due to initial drop in temperature.

**Figure 12:**
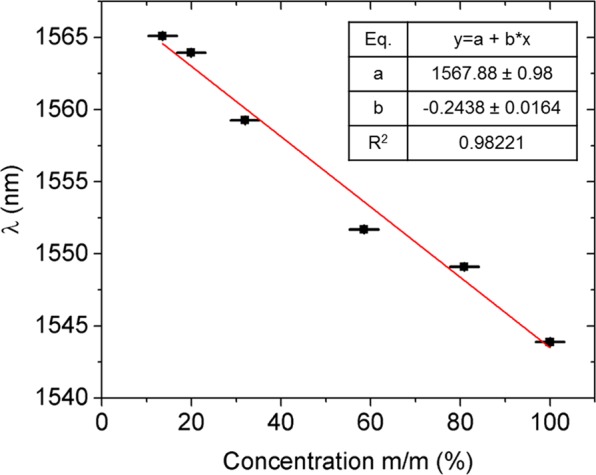
Wavelength shift of the dip position as a function of the mass concentration of chlorobenzene mixed with isopropanol.

In order to characterize the sensor sensitivity and limit of detection, the device was tested with a series of solutions featuring different mass ratios of isopropanol (IPA) and chlorobenzene. The concentration of chlorobenzene in IPA is defined as the percentage of the mass of chlorobenzene in the total mass of the solution (mass of chlorobenzene plus mass of IPA). IPA was selected to be one part of the mixture because it forms a homogeneous solution with chlorobenzene and due to both the relatively small swelling effect it induces on PDMS^14^ and its smaller refractive index difference to that of PDMS (n_IPA_ = 1.3675)^26^. When tested with pure IPA, the sensor exhibits a red shift of 0.38 nm. Assuming an IPA contribution to the overall spectral shift proportional to the percentage of IPA on the total mass of the mixture, the contribution of the IPA to the spectral shift can be offset. The corrected sensitivity of this device to chlorobenzene is extracted from a linear fit to the data in Fig. 12, yielding a spectral sensitivity of 234.8 pm/% to chlorobenzene mass percentage concentrations.

Extending the same approximation to the calculation of the refractive index of the mixture, the PDMS covered sensor exhibits sensitivity to changes of the environmental RI of 195.0 nm/RIU. This value is lower than the sensitivity obtained for the device without the PDMS cladding, as expected, since in the latter, the evanescent field interaction is directly affected by the index of the solvent in contact with the core of the waveguides. However, such loss of sensitivity is compensated by the added resilience to contamination, reusability and chemical selectivity of the proposed sensor. Higher sensitivities can be achieved by reducing the PDMS coating thickness from tens of micrometres (tested device) to about one micrometre, in consonance with the modal diameter of the supermode in the vertical axis (*z*) (Fig. 2).

Regarding the limit of detection to chlorobenzene, calculated with the IPA-offset compensated sensitivity, and considering the minimum unequivocal detectable shift to be three times the standard deviation of a set of measurements obtained from a dry stability study consisting of 20 sequential wavelength scans, the LOD was determined to be 0.24% of mass percentage concentration of chlorobenzene. As for the limit of detection in terms of volume of the analyte, the sensor successfully detected a volume as small as 200 nL, the smallest volume possible to test in our experimental setup.

**Figure 13:**
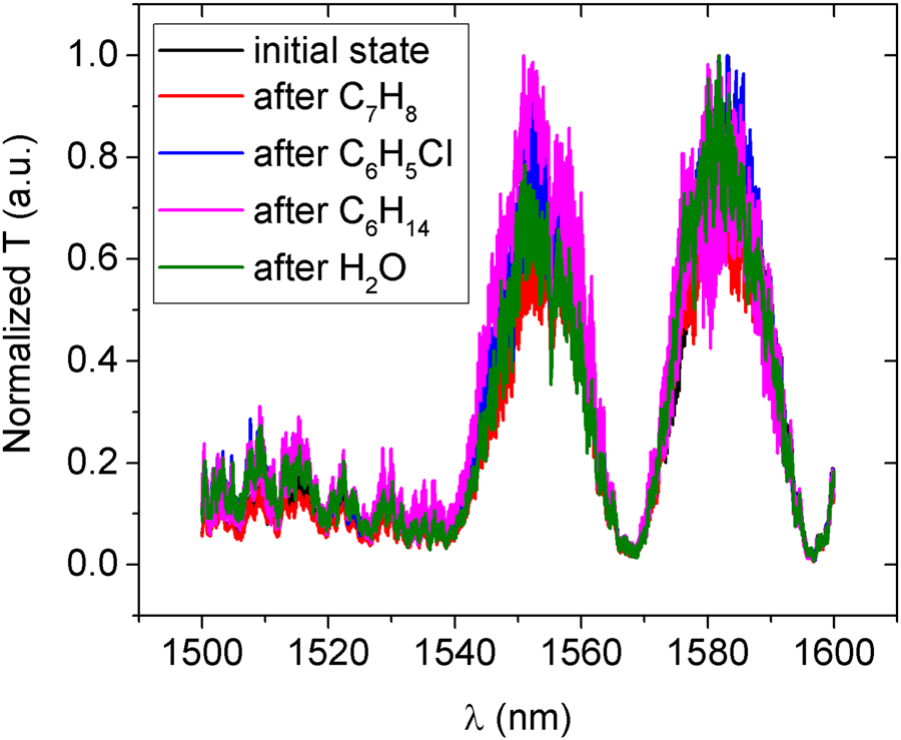
Normalized transmission output spectra of the sensor in a dry state, at different moments of the characterization process. The dry sensor spectra after undergoing testing with different chemicals (Toluene - C_7_H_8_, Chlorobenzene - C_6_H_5_Cl, Hexane - C_6_H_14_, Water - H_2_O) matches the initial dry state spectrum (prior to interaction with any solvent), highlighting the stability and sturdiness of the device under test, proving its reusable character.

The robustness and reusability of the presented sensor is illustrated in Fig. 13, representing dry measurements taken at different stages of the characterization process. The presented spectrums are normalized to their maximum value for better readability, this way accounting for different coupling and collection efficiencies due to fibre-to-chip alignment variations. It is clear that after any of the chemical tests, the sensor recovers the dry-state reference spectrum, enduring all the testing to which it was submitted including the heating-cooling cycle shown in Fig. 11, heating under vacuum (below 10 Torr), and overnight immersion in 0.5 M sulphuric acid, without any significant spectral changes, overcoming a common hindrance of optical sensors in which the cladding does not shield the devices from particles and other contaminants.

## Conclusions

The computational multiparametric study of a sensor based on multiple parallel waveguides was conducted and the corresponding prototype fabrication was carried out. The dimensions of the resulting devices did not match the designed nominal dimensions due to fabrication constrains. This mismatch supports the difference between simulations and measurements. Nonetheless, the FDTD simulated designs are still valid and invaluable tools for general device’s trends and multiparametric behaviour understanding.

The compact-size device, operating at room temperature, was experimentally tested and the validity of the concept proven as the sensor showed a selective response to changes of the environmental refractive index, being insensitivity to water but detecting the VOCs it was tested with: toluene, chlorobenzene and hexane. In addition, the sensor has confirmed the sturdiness and reliability of the concept upon multiple measurements, as the device maintained the same dry state spectrum through the entire characterization, highlighting its reusability.

A maximum spectral shift of about 22.8 nm was measured when the sensor was tested with pure chlorobenzene. The sensor was also tested with a mixture of chlorobenzene with isopropanol, from which a sensitivity of 234.8 pm/% was obtained to chlorobenzene mass percent concentrations, with a limit of detection of 0.24%_m/m_.

The presented sensor architecture displays a strong potential for transversal power distribution identification and recognition, and consequently, supports the possibility of using a less costly and fully integrated interrogation scheme through a set of monolithically integrated photodetectors at the extremity of each waveguide, further increasing its range of possible applications and field deployability.

## Data Availability

The datasets generated during and/or analysed during the current study are available from the corresponding author on reasonable request.

## References

[1] Pohanish, R. P. *Sittig’s Handbook of Toxic and Hazardous Chemicals and Carcinogens*. (Elsevier Science, 2011).

[2] Alwis KU, Blount BC, Britt AS, Patel D, Ashley DL (2012). Simultaneous analysis of 28 urinary VOC metabolites using ultra high performance liquid chromatography coupled with electrospray ionization tandem mass spectrometry (UPLC-ESI/MSMS). Analytica Chimica Acta.

[3] Dewulf J, Van Langenhove H, Wittmann G (2002). Analysis of volatile organic compounds using gas chromatography. TrAC Trends in Analytical Chemistry.

[4] Tombs M.C. (2000). VOLATILE ORGANIC COMPOUNDS IN WATER: GAS CHROMATOGRAPHY. Encyclopedia of Separation Science.

[5] Reynolds JC (2010). Detection of Volatile Organic Compounds in Breath Using Thermal Desorption Electrospray Ionization-Ion Mobility-Mass Spectrometry. Anal. Chem..

[6] Leidinger M, Sauerwald T, Reimringer W, Ventura G, Schütze A (2014). Selective detection of hazardous VOCs for indoor air quality applications using a virtual gas sensor array. Journal of Sensors and Sensor Systems.

[7] Jahangir, I. & Koley, G. Dual channel microcantilever heaters for selective detection and quantification of a generic mixture of volatile organic compounds. In *2016 Ieee Sensors* (Ieee, 2016). 10.1038/srep28735 PMC4933970 27381318

[8] Kanda K, Maekawa T (2005). Development of a WO3 thick-film-based sensor for the detection of VOC. Sensors & Actuators: B. Chemical.

[9] Stegmeier S, Fleischer M, Hauptmann P (2010). Thermally activated platinum as VOC sensing material for work function type gas sensors. Sensors and Actuators B: Chemical.

[10] Fan, Z. & Lu, J. G. Chemical sensing with ZnO nanowires. In *IEEE Sensors, 2005*. 3 pp.-, 10.1109/ICSENS.2005.1597829 (2005).

[11] Bogaerts W (2005). Nanophotonic waveguides in silicon-on-insulator fabricated with CMOS technology. Journal of Lightwave Technology.

[12] Dainesi P (2000). CMOS compatible fully integrated Mach-Zehnder interferometer in SOI technology. IEEE Photonics Technology Letters.

[13] Fain, R. *et al*. CMOS-compatible Mid-Infrared Silicon Detector. In *Conference on Lasers and Electro-Optics (2017), paper STu1N.4* STu1N.4, 10.1364/CLEO_SI.2017.STu1N.4 (Optical Society of America, 2017).

[14] Rumens CV, Ziai MA, Belsey KE, Batchelor JC, Holder SJ (2015). Swelling of PDMS networks in solvent vapours; applications for passive RFID wireless sensors. Journal of Materials Chemistry C.

[15] Kacik D, Martincek I (2017). Toluene optical fibre sensor based on air microcavity in PDMS. Optical Fiber Technology.

[16] Ning X, Yang J, Zhao CL, Chan CC (2016). PDMS-coated fiber volatile organic compounds sensors. Applied Optics.

[17] Li, Z. Y. *et al*. Highly sensitive and integrated VOC sensor based on silicon nanophotonics. In *Solid-State Sensors, Actuators and Microsystems (TRANSDUCERS), 2017 19th International Conference on* 1479–1482 (IEEE, 2017).

[18] Saunders, J. *et al*. Detection of lead contamination of water and VOC contamination of air using SOI micro-optical devices. in *Group IV Photonics (GFP), 2010 7th IEEE International Conference on* 177–179 (IEEE, 2010).

[19] Mayeh M (2009). Design and Fabrication of Slotted Multimode Interference Devices for Chemical and Biological Sensing. Journal of Sensors.

[20] Bogaerts W (2012). Silicon microring resonators. Laser & Photon. Rev..

[21] Chuang, S. L. *Physics of Photonic Devices*. (Wiley, 2009).

[22] Kedenburg S, Vieweg M, Gissibl T, Giessen H (2012). Linear refractive index and absorption measurements of nonlinear optical liquids in the visible and near-infrared spectral region. Optical Materials Express.

[23] El-Kashef, H. The necessary requirements imposed on polar dielectric laser dye solventsFII. *H. El* 4 (2002).

[24] Kerl K, Varchmin H (1995). Refractive index dispersion (RID) of some liquids in the UV/VIS between 20 °C and 60 °C. Journal of Molecular Structure.

[25] Lee JN, Park C, Whitesides GM (2003). Solvent Compatibility of Poly(dimethylsiloxane)-Based Microfluidic Devices. Anal. Chem..

[26] Sani Elisa, Dell'Oro Aldo (2016). Spectral optical constants of ethanol and isopropanol from ultraviolet to far infrared. Optical Materials.

